# Endogenous and Uric Acid-Induced Activation of NLRP3 Inflammasome in Pregnant Women with Preeclampsia

**DOI:** 10.1371/journal.pone.0129095

**Published:** 2015-06-08

**Authors:** Mariana Leticia Matias, Mariana Romão, Ingrid Cristina Weel, Vanessa Rocha Ribeiro, Priscila Rezeck Nunes, Vera Therezinha Borges, João Pessoa Araújo, José Carlos Peraçoli, Leandro de Oliveira, Maria Terezinha Peraçoli

**Affiliations:** 1 Department of Gynecology and Obstetrics, Botucatu Medical School, São Paulo State University, Botucatu, SP, Brazil; 2 Department of Microbiology and Immunology, Institute of Biosciences, São Paulo State University, Botucatu, SP, Brazil

## Abstract

Preeclampsia (PE) is a specific syndrome of pregnancy, characterized by hypertension and proteinuria. This pathology is associated with hyperuricemia and elevated serum levels of inflammatory cytokines. Uric acid crystals may activate an intracellular complex called inflammasome, which is important for processing and release of inflammatory cytokines. This study investigated the state of monocyte activation, both endogenous and stimulated with monosodium urate (MSU), by gene expression of NLRP1 and NLRP3 receptors as well as their association with inflammatory cytokines expression. Monocytes were obtained from peripheral blood of 23 preeclamptic pregnant women, 23 normotensive pregnant women (NT) and 23 healthy non-pregnant women (NP). Inflammasome activation was evaluated by the gene expression of NLRP1, NLRP3, caspase-1, IL-1β, IL-18 and TNF-α by RT-qPCR in unstimulated monocytes (endogenous expression), or after cell stimulation with MSU (stimulated expression). The concentration of cytokines was assessed by ELISA. In preeclamptic pregnant women, gene expression of NLRP1, NLRP3, caspase-1, IL-1β and TNF-α by monocytes stimulated or not with MSU was significantly higher than in NT and NP groups. Stimulation of monocytes from preeclamptic and non-pregnant women with MSU induced increased gene expression of NLRP3, caspase-1 and TNF-α in relation to the endogenous expression in these groups, while this was not observed in the NT group. The cytokine determination showed that monocytes from women with PE produced higher endogenous levels of IL-1β, IL-18 and TNF-α compared to the other groups, while the stimulus with MSU led to higher production of these cytokines in preeclamptic group than in the NT group. In conclusion, the results showed increased basal gene expression of NLRP1 and NLRP3 receptors in monocytes from PE group. These cells stimulation with MSU demonstrates that uric acid plays a role in NLRP3 inflammasome activation, suggesting the participation of this inflammatory complex in the pathogenesis of preeclampsia.

## Introduction

Preeclampsia (PE) is a specific syndrome of human pregnancy, and the leading cause of morbidity, mortality and premature delivery between 2 and 7% of pregnancies [1]. The clinical diagnosis is based on the development of hypertension (BP ≥ 140 x 90 mmHg) and proteinuria (≥300mg/24 h) [2] that occurs from the 20^th^ week of pregnancy or in the first days after birth [3].

It has been reported that PE is characterized by severe systemic inflammatory response, endothelial cell dysfunction, platelet aggregation, coagulation system activation and increased generalized vascular resistance [4, 5], which appear to contribute significantly to the pathophysiology of the disease [6]. Therefore, in PE it is observed activation of inflammatory cells such as monocytes and granulocytes, and endothelial cells [5,7], excessive production of proinflammatory cytokines [8–11] as well as lower production of regulatory cytokines such as Interleukin-10 (IL-10), and transforming growth factor (TGF-β) [12,13].

The excessive activation of intravascular monocytes/macrophages and granulocytes in PE suggests that innate immune system activation can negatively influences the pregnancy progress. Monocytes from women with PE are endogenously activated and release significantly higher concentrations of tumor necrosis factor alpha (TNF-α), superoxide anion and hydrogen peroxide compared to monocytes from normotensive pregnant women. These results confirm that PE is characterized by oxidative stress and that maternal circulating monocytes may represent an important source of free radicals and cytokines during the inflammatory disease [11,14]. Peripheral blood leukocyte activation state in pregnant women with PE is associated with genes related to inflammation [15]. There is an association between increased activation of nuclear transcription factor kappa B (NF-KB) and higher production of TNF-α and IL-1β by mononuclear cells of pre-eclamptic pregnant women as compared with normotensive pregnant women [16]. Recently, we reported that monocytes from women with PE are classically activated and produce higher levels of the inflammatory cytokines TNF-α and IL-12 associated with higher expression of TLR4 and CD64 surface markers. These results provide evidence that the systemic inflammatory environment in PE may differentiate and polarize the monocytes to the M1 phenotype [17].

Hyperuricemia has been observed in women with PE associated with the disease severity [18]. Positive correlation between increased production of TNF-α, superoxide anion and elevated serum uric acid level was observed in PE [14]. Also, high association between plasma levels of uric acid, heat shock protein 70, IL-1β, IL-12 and TNF-α has been reported in severe cases of PE [19]. Thus, high levels of uric acid in the plasma of patients with PE may represent a direct contribution to the pathogenesis of PE by its potential to promote inflammation [20] due to its association with proinflammatory cytokines.

Uric acid (monosodium urate-MSU) crystals may activate an intracellular complex called inflammasome, a multi-protein structure which is important for processing and release of IL-1β and IL-18 [21,22]. Inflammasome formation depends on receptors known as pattern recognition receptors (PRRs) expressed by cells of the innate immune system, such as monocytes, macrophages and dendritic cells [23,24]. Among the various PRRs, nod-like intracellular receptors with a pyrin domain (NLRP), are responsible for inflammasome formation in response to pathogens known as pathogen-associated molecular patterns (PAMPs) contributing to the host defense against infections [25] or also bind endogenous products of host cells, named danger-associated molecular patterns (DAMPs) [23,26,27]. Three NLR proteins have been identified to form inflammasome: NLRP1, NLRP3 and NLRC4 [22]. The NLRP3 inflammasome is the most studied inflammasome, and capable of sensing a wide variety of alarm signals [28]. In addition, some evidence indicates that NLRP3 plays an important role in inflammatory diseases such as atherosclerosis, gout, type I and type 2 diabetes [22,29,30]. After interaction with the ligand, several NLRP3 identical proteins form an oligomer and each NLRP3 of the oligomer binds to an adapter protein called ASC (apoptosis-associated speck-like protein). This binds to the inactive precursor form of caspase-1 enzyme, which becomes active and cleaves pro-IL-1β, resulting in biologically active IL-1β generation which subsequently is secreted into the extracellular medium [31,32].

Several endogenous molecules, considered “danger signals” such as MSU crystals, ATP, β-amyloid or hyaluronan) when in high concentrations, induce NLRP3 inflammasome activation, playing an important role in inflammatory diseases [27]. Conforti-Andreoni and collaborators [33] reported the importance of uric acid, together with the signaling pathway of the nuclear transcription factor-kB (NF-kB), in the inflammasome activation of dendritic cells as well as IL-1β and IL-18 production. According to Rock and collaborators [34], uric acid plays a pathophysiological role as a local alarm signal that alerts the immune system against cellular injury and aids in the development of innate and adaptive immune responses.

The NLRP3 receptor is described as uric acid sensor that induces IL-1β production in trophoblast cell lines. Thus, hyperuricemia associated to the PE could be related to activation of the NLRP3 inflammasome in trophoblast, thereby increasing the IL-1β levels in the placenta and contributing to the pathogenesis of PE [35]. Recently, a study of polimorphisms in inflammasome genes was analyzed in preeclamptic women, and showed association of NLRP1 variant rs12150220 (L155H) with development of preeclampsia, suggesting a role of this inflammasome receptor in the pathogenesis of this disorder [36].

Considering that high levels of uric acid are often observed in pregnancies complicated by PE, and are associated with oxidative stress and elevated levels of IL-1β and TNF-α produced by monocytes, the present study aimed to evaluate the role of NLRP1 and NLRP3 inflammasomes in endogenous or monosodium urate-induced activation of monocytes from pregnant women with PE. Gene expression of NLRP1 and NLRP3 receptors as well as their association with IL-1β, TNF-α and IL-18 production by these cells were determined.

## Material and Methods

### Study population

This study consisted of 46 pregnant women without previous history of hypertension or obstetric and medical complications, admitted to the Obstetric Unit of Botucatu Medical School, Sao Paulo State University, Botucatu, SP, Brazil between March 2013 and October 2014. Twenty three women were diagnosed with preeclampsia (PE), defined as a persistent elevated blood pressure value of 140x90 mmHg and proteinuria (≥ 300 mg in urine collected during 24 hours) after the 20^th^ weeks of gestation [2], and 23 pregnant women with an uncomplicated pregnancy that remained normotensive (NT) and nonproteinuric throughout pregnancy were recruited as controls and matched for gestational age with the preeclamptic group. A group of 23 non-pregnant women (NP) was added to establish the comparison of immunological parameters between groups. Gestational age was calculated from the last menstrual period and confirmed by ultrasound dating. Non-pregnant women were voluntary donors of the Blood Bank of the Hemocenter of the Botucatu Medical School, Botucatu, SP, Brazil. Proteinuria in 24-hour urine was measured by a colorimetric method, the Technicon RAXT automation system, and uric acid was assessed by uric acid enzymaticTrinder (Biotrol Diagnostic) inthe Clinical Laboratory, Botucatu Medical School, Botucatu, SP, Brazil. Exclusion criteria for the three groups included chronic hypertension, multiple gestation, prior preeclampsia, illicit drug use, and preexisting medical conditions such as diabetes, cancer, acute infectious disease, cardiovascular, autoimmune, renal and hepatic diseases. All of the patients and controls agreed to participate in the study after due clarification and signing of a written informed consent form. This study was approved by Botucatu Medical School–UNESP Research Ethics Committee.

### Blood sampling

The whole blood for evaluation of gene expression and cytokine production by monocytes from pregnant women with PE was collected at the time of disease diagnosis, and from normotensive pregnant women at the time they were matched for gestational age with pregnant women with PE. The blood of non-pregnant women was obtained during blood donation. Blood samples obtained from the antecubital vein were put into a plastic tube containing 5% EDTA.

### Isolation and culture of monocytes

Peripheral blood mononuclear cells (PBMCs) were isolatedby density gradient centrifugation on Ficoll-Paque Premium [density (d) = 1.077] (GE Healthcare Bio-Sciences, Uppsala, Sweden) according to the method described by Boyum [37]. Briefly, 10 mL of blood was mixed with an equal volume of RPMI 1640 tissue culture medium (Sigma-Aldrich, Chemical Co., St Louis, Missouri, USA) containing 2 mM L-glutamine, 10% heat-inactivated fetal bovine serum, 20 mM HEPES, and 40 μg/mL gentamicin (complete medium). Samples were layered over 5 mL Ficoll-Paque Premium in a 15 mL conical plastic centrifuge tube. After centrifuging at 400 g for 30 minutes at room temperature, the interface layer of PBMC was carefully aspirated and washed twice with phosphate-buffered saline (PBS) containing 0.05 mM ethylenediaminetetraacetic acid (PBS-EDTA) and once with complete medium, with centrifugation in between washes at 300 g for 10 minutes. Cell viability, as determined by 0.2% Trypan Blue dye exclusion, was >95% in all experiments. Monocytes were counted using neutral red (0.02%) in PBMC suspension and a suspension of 5x10^5^ monocytes/mL in complete medium was employed for gene expression and cytokines determination.

### Monocyte culture supernatants

Monocyte suspensions (5x10^5^ cells/mL) were distributed (1 mL/well) in 24-well flat-bottomed plates (Greiner Bio-One, Germany). After incubation for 2 h at 37°C in a humidified 5% CO_2_ atmosphere, non-adherent cells were removed by aspiration and each well rinsed twice with complete medium. Monocyte preparations routinely contained >90% monocytes as determined by morphologic examination and staining for nonspecific esterase [38]. Monocytes were incubated with complete medium in the presence or absence of 50μg/mL of monosodium urate (MSU) (Invivogen, San Diego, CA, USA) for 18 h at 37°C in 5% CO_2_.The MSU concentration used as stimulus in monocyte cultures was previously standardized employing monocytes from five health non-pregnant women.This concentration of MSU (50 μg/mL) was chosen to simulate the levels of uric acid in preeclamptic women plasma, ranging from 45 to 100 μg/mL. Culture supernatants were harvested and stored at -80°C for cytokine determination.

### Cytokine determination

Cytokine concentrations in monocyte culture supernatants were determined by enzyme-linked immunosorbent assay (ELISA), using Quantikine ELISA kits (R&D Systems, Minneapolis, MN, USA) for TNF-α and IL-1β according to the manufacturer’s instructions. Assay sensitivity limits were 1.6 pg/mL for TNF-α and 1.0 pg/mL for IL-1β. For IL-18 quantification a quantitative test ELISA kit for human IL-18 (MBL–Medical & Biological Laboratories, Nagoya, Japan) with sensitivity of 12.5 pg/mL was employed.

### Evaluation of the expression of transcripts related to inflammation

Monocytes from normotensive, preeclamptic and non-pregnant women were incubated with complete medium in the presence or absence of 50 μg/mL of monosodium urate (MSU) (Invivogen, San Diego, CA, USA) for 4 h at 37°C in 5% CO_2_, and were subjected to analysis of the expression, on the transcriptional level, of the gene encoding the proteins NLRP1, NLRP3, caspase-1, IL-1β, IL-18 and TNF-α. Glybenclamide (Sigma-Aldrich, St Louis, MO, USA) at concentration of 50 and 200μM was added to cultures of monocytes from non-pregnant women for 30 min, and then MSU (50 and 100 μg/mL) was added to the culture for another 4h in order to evaluate its inhibitory effect on NLRP3 inflammasome activation by MSU. Total RNA was extracted from monocytes through the system Total RNA Purification Kit (Norgen Biotek Corp., Thorold, Canada) according to manufacturer's protocol. After extraction, to ensure complete removal of genomic DNA, 1 μg of total RNA was incubated with DNAse I Amp Grade (Invitrogen). The purity and relative quality of all samples of total RNA obtained were determined by fluorometry using the equipment Qubit Fluorometric Quantitation (Life Technologies). Subsequently, the synthesis of complementary DNA (cDNA) for performing the polymerase chain reaction coupled reverse transcription (Reverse Transcription-coupled polymerase chain reaction—RT-PCR) was conducted with 450ng of total RNA in 60μL reaction using ImProm- IITM Reverse Transcription System, according to manufacturer's protocol.

The quantification of gene expression of NLRP1, NLRP3, caspase-1, IL-1β, IL-18 and TNF-α was made through the reaction technique in quantitative Polymerase Chain in real time (RT-qPCR) using RT GoTaq-qPCR Master Mix (Promega, Madison, WI, USA). The device used was 7500 Fast Real Time PCR Systems (Applied Biosystems, USA).

The variants of the targets studied were aligned in the MEGA 5.1 program and subsequently each primer was selected by the software Primer-BLAST. Primers located in exon-exon junction guarantee the purity of the reaction, namely the absence of any genomic DNA that may contaminate it. The primer sequences used in this study are in Table 1.

**Table 1:** Primers for inflammasomes proteins, cytokines and GAPDH.

Gene	Forward primer (5’- 3’)	Reverse primer (5’– 3’)	GeneBank
NLRP1	(1728)TCCGGCTCCCATTAGACAGA(1747)	(1810)AGACCCATCCTGGCTCATCT(1791)	NM_033004.3
NLRP3	(2826)GAGGAAAAGGAAGGCCGACA(2845)	(2917)TGGCTGTTCACCAATCCATGA(2897)	NM_004895.4
Caspase-1	(1065)AGACATCCCACAATGGGCTC(1084)	(1172)TGAAAATCGAACCTTGCGGAAA(1151)	NM_033292.3
IL-1β	(544)GAGCAACAAGTGGTGTTCTCC(564)	(653)AACACGCAGGACAGGTACAG(634)	NM_000576.2
IL-18	(438)ACTGTAGAGATAATGCACCCCG(459)	(517)AGTTACAGCCATACCTCTAGGC(496)	NM_001562.3
TNF-α	(325)GCTGCACTTTGGAGTGATCG(344)	(462)GGGTTTGCTACAACATGGGC(443)	NM_000594.3
GAPDH	(684)CGTGGAAGGACTCATGACCA(703)	(801)GGCAGGGATGATGTTCTGGA(782)	NM_002046.4

Each reaction was set in duplicate in a total of 20μL each, which contains 0,3 μM of each primer (forward and reverse), 2 μL of cDNA, 10 μL of master mix and 6 μL of nuclease-free water. Additionally, was inserted a control, also in duplicate, which was included in each reaction in order to prove that there is no contamination. The conditions for the RT-qPCR reactions were: initial denaturation at 96°C—2 min and then 40 cycles at 95°C- 15s and 60°C -60s, followed by a melting curve. The amplification of each particular transcript was confirmed by melting curve generated profile of the end of each reaction.

Expression values of the analyzed transcripts were normalized based on the concurrent analysis of the expression of the enzyme encoding glyceraldehyde-3-phosphate dehydrogenase gene (GAPDH). The calculation of the differential expression of selected genes was carried out by the data processing method compared to a standard curve [39]. All conditions, including GAPDH for each sample and the negative control reaction (No Template Control—NTC, absence of RNA) were analyzed in duplicates. To analyze the relative expression, after the analysis of gene expression, we chose an RNA sample obtained from each group, which received the relative value of 100. All other samples received values for that sample.

### Statistical analysis

Statistical significance was evaluated by the non-parametric methods, Kruskal-Wallis test and Mann-Whitney *U* test, using the statistical program INSTAT, Graph Pad, San Diego, California. Differences between the groups studied were considered statistically significant when the p<0.05.

## Results

### Demographic and clinical characteristics

No difference in age, gestational age, race and parity parameters among preeclamptic, normotensive and non-pregnant women was observed (Table 2). Systolic and diastolic blood pressure as well as serum uric acid concentration were significantly higher in pregnant women with PE compared with normotensive pregnant and non-pregnant women. Additionally, proteinuria levels were significantly higher in pregnant women with PE than in normotensive pregnant women.

**Table 2:** Demographic and clinical characteristics of pregnant women with preeclampsia, normotensive pregnant women and non-pregnant women.

Characteristics		Pregnant women with preeclampsia (n = 23)	Normotensive pregnant women (n = 23)	Non-pregnant women (n = 23)
**Age (years)**		25 (14–41)	25 (13–43)	24 (21–42)
**Race**	**White (%)**	84.6	91.3	87.0
	**Non-white(%)**	15.4	8.7	13.0
**Gestational age (weeks)**		35 (24–40)	34 (23–39)	N/A
**Parity**	**Nulliparous (%)**	63	68	N/A
	**Multiparous (%)**	37	32	
**Systolic Blood Pressure (mmHg)**		160 ^#^ (140–200)	110 (90–112)	114 (100–120)
**Diastolic Blood Pressure (mmHg)**		110 ^#^ (90–120)	69 (63–70)	70 (65–80)
**Proteinuria (mg / 24h)**		1540 * (300–19 880)	< 300	N/A
**Uric acid (mg/dL)**		5.9 ^#^ (4.5–10.1)	3.8 (2.2–4.6)	4.1 (2.8–4.8)

Data are presented as median, with the minimum and maximum values in parentheses, or as percentage. N/A = not applicable.

* (p <0.05) vs normotensive pregnant women (Mann-Whitney *U* test)

# (p <0.05) vs normotensive pregnant women and non-pregnant women (Kruskal-Wallis test).

### Expression of genes related to inflammasome in monocytes from pregnant and non-pregnant women

**Fig 1:**
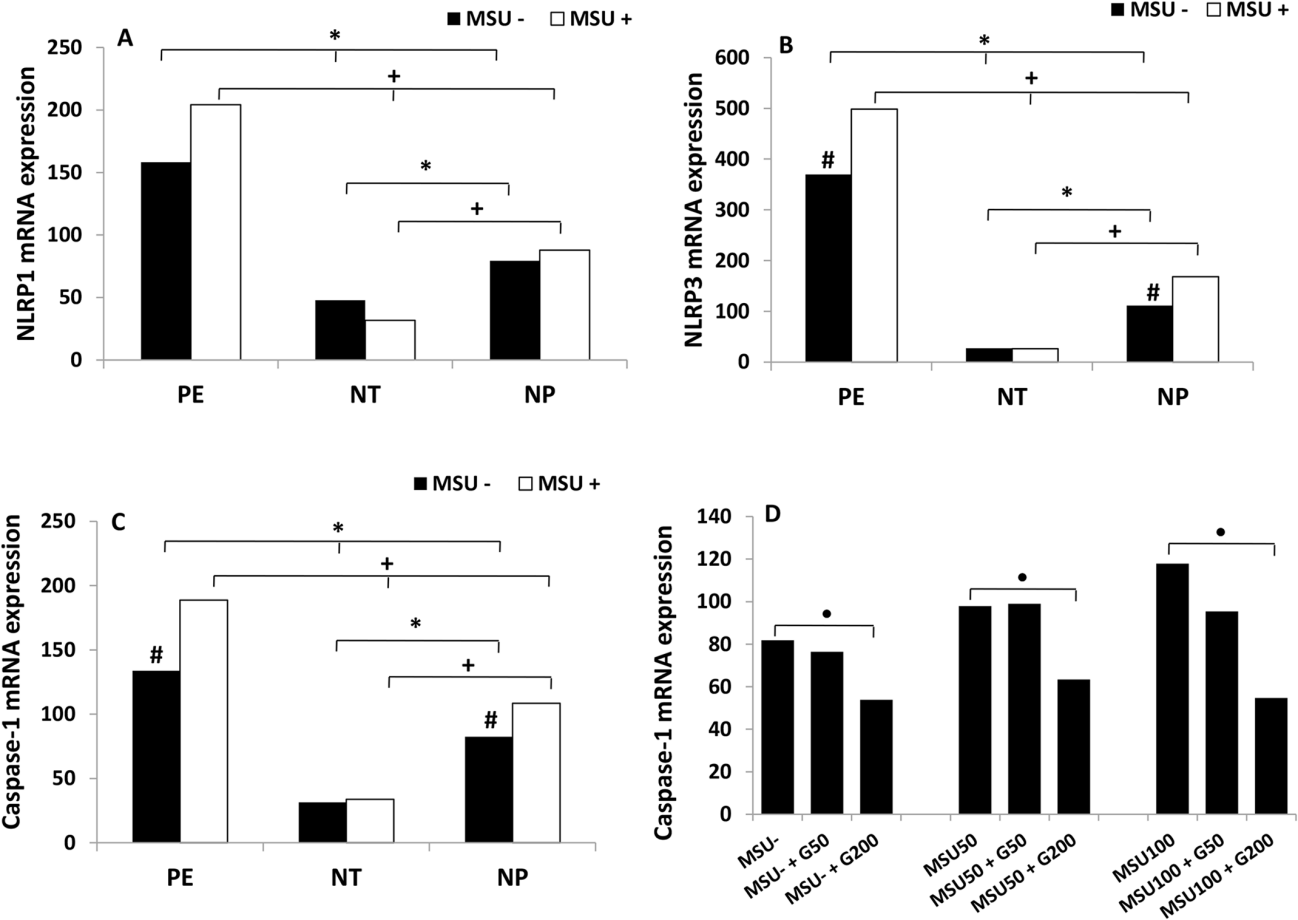
Gene expression of NLRP1 (A), NLRP3 (B) and caspase-1 (C) by monocytes. Monocytes from pregnant women with preeclampsia (PE), normotensive pregnant women (NT) and non-pregnant women (NP), were cultured in the presence (MSU^+^) or absence (MSU^-^) of monosodium urate. Expression of caspase-1 (D) by monocytes from non-pregnant women treated with 50 μM or 200 μM of Glybenclamide and cultured in the presence (MSU^+^) or absence (MSU^-^) of monosodium urate. Results are shown as median. *P<0.*05* shows significant difference between MSU^-^ groups; ^+^ *P<0*.*05* shows significant difference between MSU^+^ groups; # *P<0*.*05vs*MSU^+^; ^●^ *P<0*.*05*(MSU^-^ *vs*MSU^-^ + G200), (MSU50 *vs* MSU50+G200), (MSU100 *vs* MSU100+G200).

Fig 1A, 1B and 1C show, respectively, higher gene expression of NLRP1, NLRP3 receptors and caspase-1 in monocytes from pregnant women with PE, stimulated or not with MSU, when compared to NT and NP groups. Lower expression of these genes was detected in monocytes from normotensive pregnant women both stimulated or not with MSU, compared to NP group. In preeclamptic pregnant and NP groups, MSU induced higher expression of NLRP3 receptor and caspase-1 compared to endogenous levels of this mRNA in monocytes from these groups. There was a tendency to an increase in NLRP1 mRNA expression in cells of pregnant women with PE stimulated with MSU compared with non-stimulated cells, but this difference was not significant.

The results also showed that thetranscriptional profile of NLRP3 receptor was more elevated than the NLRP1 one, in monocyte from pregnant women with PE and in non-pregnant women. Fig 1D shows that Glybenclamide at 200 uM significantly inhibited caspase-1 mRNA expression by monocytes from non-pregnant women in a dose-dependent manner.

**Fig 2:**
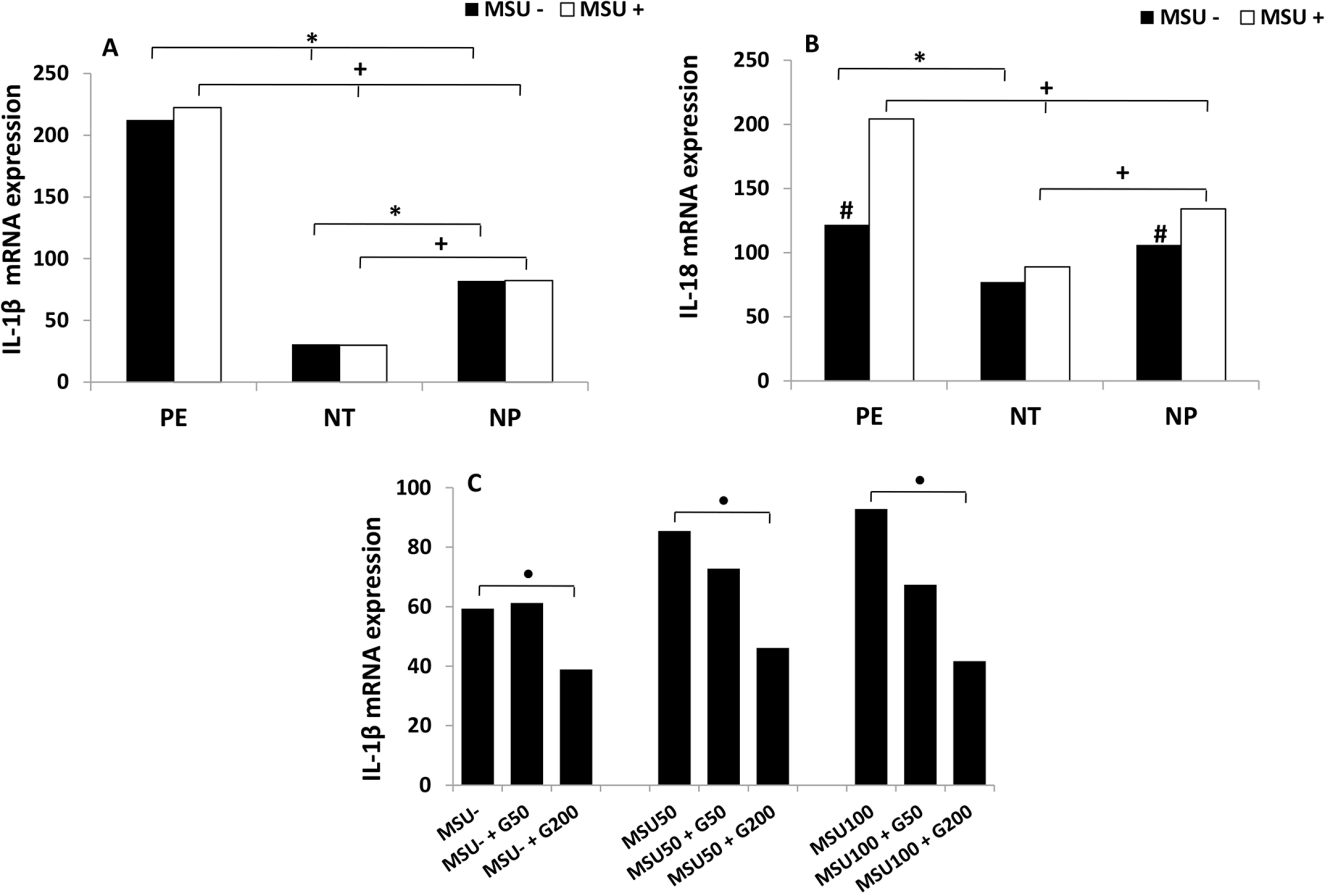
Gene expression of IL-1β (A) and IL-18 (B) by monocytes. Monocytes from pregnant women with preeclampsia (PE), normotensive pregnant women (NT) and non-pregnant women (NP), were cultured in the presence (MSU^+^) or absence (MSU^-^) of monosodium urate. Expression of IL-1 β (C) by monocytes from non-pregnant women treated with 50 μM and 200 μM of Glybenclamide, in the presence (MSU^+^) or absence (MSU^-^) of monosodium urate. Results are shown as median.*P<0.*05* shows significant difference between MSU^-^ groups; ^+^ *P<0*.*05* shows significant difference between MSU^+^ groups; # *P<0*.*05vs*MSU^+^; ^●^ *P<0*.*05* (MSU^-^ *vs*MSU^-^ + G200), (MSU50 *vs* MSU50+G200), (MSU100 *vs* MSU100+G200).

Fig 2A represents higher gene expression of IL-1β in monocytes from pregnant women with PE, stimulated or not with MSU, when compared to NT and NP groups. On the other hand, lower expression of IL-1β gene is observe in monocytes from normotensive pregnant women, stimulated or not with MSU, compared to NP group. There was no difference between IL-1β mRNA levels from endogenous and stimulated with MSU monocytes cultures for the three groups. Glybenclamide added to monocyte cultures from non-pregnant women, in the presence or absence of MSU significantly inhibited IL-1β mRNA expression in a dose-dependent fashion (Fig 2C). Inhibitory effect was achieved with the concentration of (200μM).

Endogenous gene expression of IL-18 in monocytes from preeclamptic pregnant women was significantly higher than in the NT group (Fig 2B). After MSU stimulation monocytes from women with PE expressed higher IL-18mRNA as compared to NT and NP groups. Cells from NT group stimulated with MSU had lower gene expression of this cytokine in relation to NP group. In addition, IL-18 gene expression in monocytes from pregnant women with PE stimulated with MSU (MSU+) was significantly higher compared to non-stimulated cells (MSU-).

**Fig 3:**
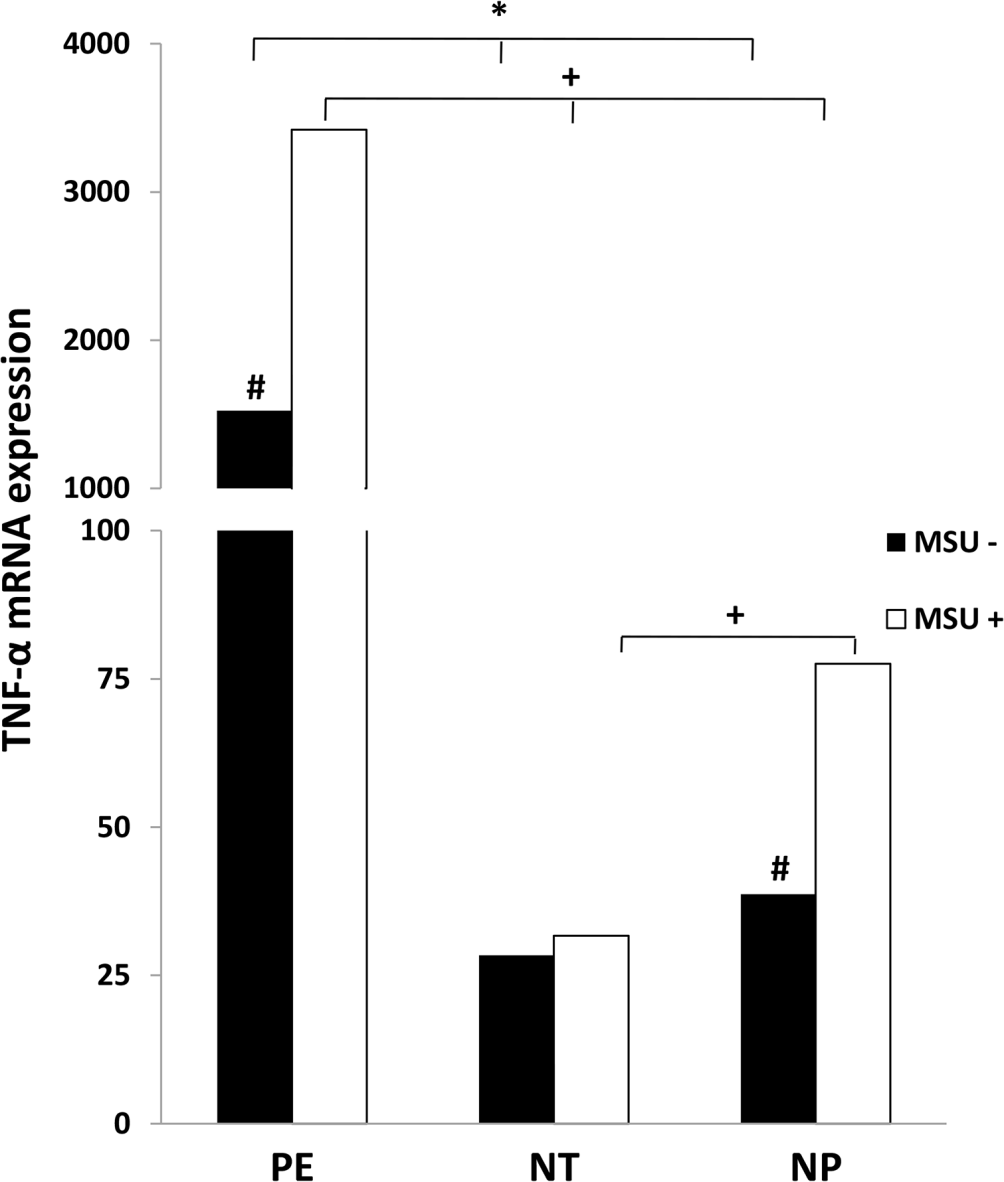
Gene expression of TNF-α by monocytes. Monocytes from pregnant women with preeclampsia (PE), normotensive pregnant women (NT) and non-pregnant women (NP), were cultured in the presence (MSU^+^) or absence (MSU^-^) of monosodium urate. Results are shown as median.*P<0.*05* shows significant difference between MSU^-^ groups; ^+^ *P<0*.*05* shows significant difference between MSU^+^ groups; # *P<0*.*05 vs* MSU^+^.

Fig 3 shows anintense increase in gene expression of TNF-α in monocytes from pregnant women with PE, cultured with and without MSU, when compared to NT and NP groups. In normotensive pregnant women group, the stimulation with MSU did not alter the lower expression of TNF-α by monocytes compared to NP group. Furthermore, there is significant difference between pregnant women with PE and non-pregnant women in relation to TNF-α gene expression in monocytes stimulated or not with MSU.

### Cytokine production by monocytes from pregnant and non-pregnant women

**Fig 4:**
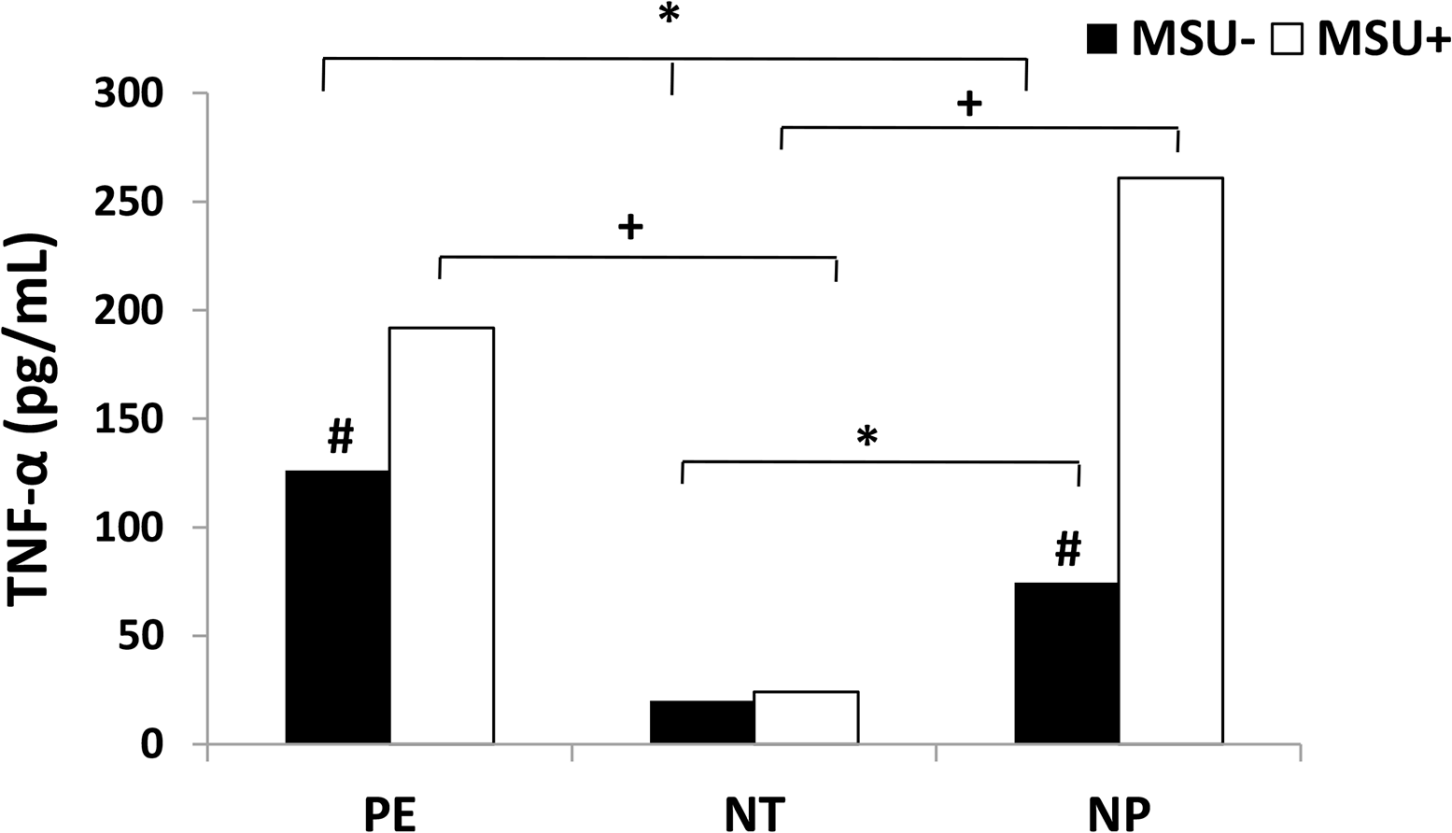
IL-1β (A) and IL-18 (B) production by monocytes. Monocytes from pregnant women with preeclampsia (PE), normotensive pregnant women (NT) and non-pregnant women (NP), were cultured in the presence (MSU^+^) or absence (MSU^-^) of monosodium urate. Results are shown as median. *P<0.*05* shows significant difference between MSU^-^ groups; ^+^ *P<0*.*05* shows significant difference between MSU^+^ groups; # *P<0*.*05 vs* MSU^+^.

**Fig 5:**
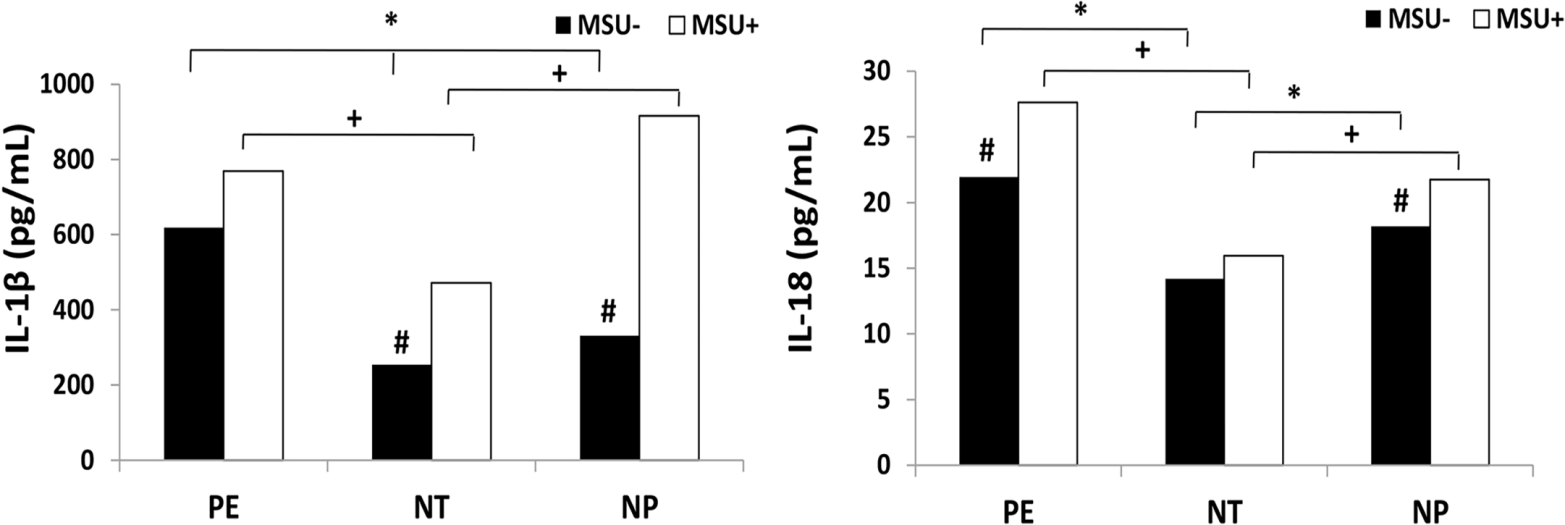
TNF-α production by monocytes. Monocytes from pregnant women with preeclampsia (PE), normotensive pregnant women (NT) and non-pregnant women (NP), were cultured in the presence (MSU^+^) or absence (MSU^-^) of monosodium urate. Results are shown as median. *P<0.*05* shows significant difference between MSU^-^ groups; ^+^ *P<0*.*05* shows significant difference between MSU^+^ groups; # *P<0*.*05 vs* MSU^+^.

The production of IL-1β, IL-18 and TNF-α cytokines in monocytes from 23 pregnant women with PE, 23 normotensive pregnant women and 23 healthy non-pregnant women, stimulated or not with monosodium urate (MSU) is shown in Figs 4A, 4B and 5 respectively.

There is an increase in the endogenous concentration of IL-1β produced by monocytes from pregnant women with PE compared to NT and NP groups. The MSU stimulation induced higher production of IL-1β by cells from preeclamptic pregnant women compared to NT group. It is also possible to notice a decrease in cytokine expression in monocytes from normotensive pregnant women stimulated with MSU compared to the NP group (Fig 4A). However, there is significant difference between the production of IL-1β by monocytes from NT groups and NP that were stimulated with MSU (MSU+) and the basal production of these cells. There was a tendency to increase levels of IL-1β produced bymonocytes from the preeclamptic group, when the cells were stimulated with MSU compared to unstimulated cells, but this difference did not reach statistical significance.

Fig 4B shows a significant increase in endogenous and MSU-stimulated protein concentration of IL-18 produced by monocytes from pregnant women with PE, compared with the NT group. Monocytes from NT group, cultured with or without MSU produced lower levels of this cytokine compared to the NP Group. In addition, there was significant difference between endogenous and MSU-stimulated IL-18 production both in preeclamptic and NP groups.

In Fig 5 it is observed higher endogenous levels of TNF-α produced by monocytes from pregnant women with PE in relation to NT and NP groups. The MSU stimulation led to increased TNF-α production by preeclamptic group only when compared to the NT group. It is also observed lower production of TNF-α protein by monocytes from NT group, stimulated or not with MSU, in comparison to NP group. Monocytes from preeclamptic pregnant and non-pregnant women stimulated with MSU showed higher TNF-α production in comparison to the basal levels produced by these cells.

## Discussion

In pregnant women with PE elevated serum levels of uric acid associated with increased production of pro-inflammatory cytokines such as IL-1β and TNF-α by mononuclear cells from peripheral blood [16], suggest participation of hyperuricemiain the pathogenesis of PE. It is known that uric acid crystals (MSU) act as DAMPs activating the complex NLRP3 inflammasome [40]. The results of the present study show higher gene expression of NLRP1 and NLRP3 receptors, caspase-1 as well as IL-1β, IL-18 and TNF-α in peripheral blood monocytes of pregnant women with PE. These results suggest the endogenous activation of these cells, associated with increased production of inflammatory cytokines compared with monocytes from normotensive pregnant women.

It was observed that the gene expression of NLRP1, NLRP3 and caspase-1 have similar profiles to each other and higher expression in monocytes from pre-eclamptic women compared with normotensive pregnant women and non-pregnant women groups. A recent study [35], showed that uric acid activates the inflammasome in trophoblast cells, leading to IL-1β secretion and, suggesting that it is a novel mechanism for induction of inflammation in the maternal-fetal interface and causing adverse pregnancy outcomes, including preeclampsia. Caspases are important cysteinyl aspartate proteases in apoptosis and inflammation mechanisms [41]. Caspase-1 is an important inflammatory protein synthesized as pro-caspase-1, and becomes biologically activated when inflammasome starts its generation process [42,43]. It is recognized in the literature that NLRP1, NLRP3, AIM2 and IPAF inflammasomes are responsible for caspase-1 activation process, leading to the processing and release of IL-1β and IL-18 [44].

According to Martinon et al. [21], monosodium urate triggers the increase in gene expression of NLRP3 and caspase-1 in lineage of THP1 monocytes. Our results corroborate with these authors, showing that the stimulus with MSU induces increased NLRP3 and caspase-1 expression in monocytes from women with PE and from non-pregnant women, while the NLRP1 gene showed only a tendency to increased expression by monocytes of pregnant women with PE without statistical significance. Our results about reduction of gene expression of caspase-1 and IL-1β by monocytes stimulated with MSU in the presence of Glybenclamide, an inflammasome inhibitor [45] suggests that caspase-1 and IL-1β expression are dependent on NLRP3 activation by MSU. However, the precise molecular details of NLRP3 inflammasome in response to uric acid in preeclampsia remain to be elucidated. In the present study, the demonstrated capacity of NLRP3 inflammasome in inducing IL-1β production suggests a role for this mechanism in the systemic inflammatory response observed in preeclampsia.

The gene expression of NLRP1 and NLRP3 receptors, caspase-1, IL-1β, IL-18 and TNF-α, as well as the production of these cytokines was higher in monocytes from pregnant women with PE stimulated with MSU compared with normotensive pregnant women.

However, when compared to monocytes from healthy women, this increase is significant only in transcriptional, but not in protein level. This difference between gene expression and production of cytokines could be related to the fact that multiple ribosomes can translate a single mRNA molecule at the same time. Ribosomes groups called polysome, allow the simultaneous production of multiple strings of amino acids (polypeptides). These polypeptides may be complete or require further processing to become mature and to acquire a three-dimensional conformation [46].

The production of IL-1β, IL-18 and TNF-α by monocytes from non-pregnant women stimulated with MSU was increased, reaching a maximum level of production, similar to that observed in pregnant women with PE. These results confirm the ability of uric acid in exerting stimulatory effect on inflammatory activity of these cells by inflammasome activation represented by high expression of the genes encoding the NLRP3 receptor and caspase-1 protein, as well as increase in the inflammatory cytokines IL-1β and IL-18 production after cell stimulation in NP group.

In preeclamptic women there was no significant increase in gene expression and production of IL-1β after monocyte stimulation with MSU as showed in Fig 2. This phenomenon may be occurred probably because the cells were already significantly activated in PE, and producing higher endogenous levels of this cytokine. Siljee et al. [47] suggest that increased serum levels of IL-1β during the first trimester of pregnancy, in the absence of other inflammatory proteins, can be considered an early biomarker of PE onset, and an important pathogenic factor for the development of this disease.

Monocytes from normotensive pregnant women stimulated with MSU, in turn, have lower gene and protein expression of inflammatory cytokines IL-1β, IL-18 and TNF-α in relation to PE and NP groups. The lowest gene expression of these cytokines by monocytes from NT group could be due to the regulatory activity of IL-10 on these cells. The predominance of higher levels of this anti-inflammatory cytokine predominance in normal pregnancy acts to minimize the deleterious effects of excessive inflammatory response, being able to regulate the inflammatory response that occurs during pregnancy by controlling the IL-1β and TNF-α gene expression [48]. It is known that IL-10 exerts a potent inhibitory effect on the production of inflammatory cytokines such as IL-1β and TNF-α by activated monocytes [49]. However, in PE, this regulation does not occur adequately, and the balance is altered, with decreased IL-10 and increased TNF-α [50]. In previous work, it was shown that monocytes from preeclamptic pregnant women produce endogenous IL-10 significantly lower than in normotensive pregnant women, while TNF-α values are high [13], confirming the findings in the literature [12,51].

In the present study no significant differences were detected between preeclamptic and NP groups in relation to endogenous IL-18 gene expression and cytokine production. However, monocytes from these both groups produced higher levels of IL-18 after MSU stimulation (Figs 2B and 4B). Pro-IL-18 is constitutively present in healthy people monocytes [52]. Then, it was expected that the increase in gene and protein expression was not as obvious as that of other inflammatory cytokines. IL-18 is also secreted by inflammasome activation [53,54]. We also observed that there was more intense production of IL-1β than IL-18, demonstrated by high levels of IL-1β secreted by monocytes from the three groups. It is believed that this may be occurred because IL-18 also stimulates the secretion of Th2 cytokines with anti-inflammatory activity [55]. Thus, IL-18 induces Th1 and Th2 responses, acting as a cytokine with both inflammatory and anti-inflammatory profiles.

The production of proinflammatory cytokines is a critical step for an effective innate immune response and is also a mechanism by which this response influences the subsequent development of adaptive immune response [56]. IL-1β and IL-18 are examples of key cytokines which not only activates monocytes, macrophages and neutrophils but also specifically direct the development of the adaptive immune response of CD4+ T cells in humans and mice. The differentiation of CD4+ T cells in the presence of IL-1β and IL-18 results in Th17 and Th1 effector cells, respectively [57,58]. The predominant profile of Th17 cells on regulatory T cells in pregnant women with PE could act by modulating the Th1/Th2 balance [59], suggesting that both innate and adaptive immunity are involved in the excessive inflammatory response that occurs in this obstetric pathology [60].

The endogenous and MSU-induced gene expression of TNF-α, was higher in monocytes from pregnant women with PE compared to NT and NP groups, while the cytokine production was significantly higher in PE group than in the NT group. This increased TNF-α production by monocytes from pre-eclamptic pregnant women confirms previous results from the literature [11,13, 61], demonstrating that elevated serum uric acid levels correlate with increased production of TNF-α and free radicals released by monocytes from pregnant women with PE compared to normotensive pregnant women [14]. These elevated levels of TNF-α in peripheral blood of pre-eclamptic pregnant woman might activate NLRP1 and NLRP3 inflammasomes in monocytes by its stimulatory effect on the nuclear factor-κB transcription. The synthesis of TNF-α and IL-1β is controlled, in part, by NF-κB, which is more active in pregnant women with PE cells [16]. NF-κB regulates the transcription of genes related to inflammation [62,63] and TNF-α, in turn, acts stimulating NF-κB activation, and maintaining a cell cycle activation [64]. Thus, although not directly related to the inflammasome, TNF-α is an important inflammatory cytokine produced in the first activation signal of this multiprotein complex.

Activation of inflammasomes by MSU requires two signals. The first is the recognition of MSU by the extracellular toll-like (TLR) receptor, leading to the nuclear transcription factor (NF-κB) activation, and causing production of pro-IL-1β by monocytes, as previously described in gout [22]. The second signal depends on phagocytosis of MSU crystals and their recognition by NLRP3 inflammasome in monocytes, resulting in activation of caspase-1 and subsequent cleavage of pro-IL-1β to produce active IL-1β. According to Bauernfeind et al. [65], the need for this double stimulation to activate the inflammasome might be important for preventing uncontrolled activation of NLRP3, a responsible that could lead to devastating consequences in the host, as seen in autoinflammatory diseases.

In conclusion, the present results show, for the first time, that monocytes from preeclampticwomen have endogenous activation of the inflammasomes NLRP1 and NLRP3 and secrete higher levels of IL-1β, IL-18 and TNF-α. Stimulation of these cells with MSU, inducesincrease in gene expression of NLRP3 receptor and caspase-1, as weel as IL-18 production, that is more evident in pre-eclamptic pregnant and non-pregnant women groups. These results demonstrate the role of uric acid in NLRP3 inflammasome activation and release of inflammatory cytokines, suggesting the envolvement of this inflammatory complex in the pathogenesis of preeclampsia.
